# Diabetes in US women on the rise independent of increasing BMI and other risk factors; a trend investigation of serial cross-sections

**DOI:** 10.1186/1471-2458-14-954

**Published:** 2014-09-15

**Authors:** Adaeze Ibe, Tyler C Smith

**Affiliations:** Department of Community Health, School of Health and Human Services, National University Technology and Health Sciences Center, 3678 Aero Court, 92123 San Diego, CA USA

## Abstract

**Background:**

The epidemic of diabetes continues leaving an enormous and growing burden of chronic disease to public health. This study investigates this growing burden of diabetes independent of increasing BMI in a large population based female sample, 2006–2010.

**Methods:**

Serial cross-sectional data using the Behavioral Risk Factor Surveillance System (BRFSS) 2006–2010 surveys from 1,168,418 women. Diabetes was assessed by self-report of a physician diagnosis, and body mass index (BMI) was calculated based on self-reported height and weight.

**Results:**

Almost 60% of women responders had a BMI > 25 (defined as overweight or obese). Diabetes was reported in 16% of respondents whose BMI > 25, and in 4% of respondents with reported BMI ≤ 25. Overall, 11% of the women in this sample reported being diagnosed with diabetes, of whom 83% had a BMI > 25. BMI, physical activity, age, and race were each independently associated with diabetes (*p*-value < 0.05). The odds of reported diabetes increased each year independent of BMI, physical activity, age, and race.

**Conclusions:**

After adjusting for age, race, physical activity, and year of survey response, results indicate a threefold increase in diabetes among respondents with a BMI > 25 (OR = 3.57; 95% CI = 3.52-3.63). Potentially more alarming was a notable increase in odds of diabetes across the years of study among women, implying a near 30 percent projected increase in odds of diabetes diagnoses by 2020. This is likely due to advances in diagnosis and treatment but also highlights a burden of disease that will have a growing and sustained impact on public health and healthcare systems.

## Background

Type 2 diabetes is an epidemic in the United States with more than eight percent, or roughly 25.8 million people currently diagnosed [1]. This debilitating disease is strongly associated with many chronic diseases such as heart disease, respiratory diseases, hypertension, osteoporosis, kidney failure, nervous system diseases, pregnancy complications, non-traumatic lower limb amputations, biochemical imbalances, and retinal problems [1–5]. More so, the risk of death is twice as high in those with diabetes as without and has been identified as one of the top 10 causes of death in the United States [1, 6]. Studies have shown that people with prediabetes who lose weight and increase their physical activity can prevent or delay diabetes and to a large extent stem the risk of developing heart disease and stroke [1, 7]. Lifestyle changes explained by the epidemiological transition have been largely cited as reasons for increased prevalence of obesity and diabetes over recent years [8, 9] and a projected increase in prevalence is due to known factors such as an aging population, an increase in high-risk minority populations, and longer life expectancies of people diagnosed with diabetes [10].

Women, particularly ethnic minority women, are at higher risks of diabetes and associated complications, and contribute to current and future increases in rates of healthcare utilization [11]. Diabetes affects every stage of women’s lives –from childhood (type 1 diabetes) to the reproductive and child-bearing ages (gestational diabetes) and leading up to the onset of type 2 diabetes in adolescent, middle aged and older women [4, 11].

Much focus has been given to the risk factors (a sedentary lifestyle, body mass index, age, ethnicity [12, 13], that are contributing to increase in incidence of diabetes especially in female populations. However, little is known about the projected prevalence of diabetes in high risk populations even if there were a reduction in risk factors. Because of the enormous implications to the burden of disease and associated chronic diseases and thus the resources needed to manage this health epidemic, the objective of this study was to assess a population level change in diabetes prevalence among women over a 5-year period independent of known risk factors that may be themselves influencing the increase in the burden of diabetes.

## Methods

### Data

The analyses utilized data from the Behavioral Risk Factor Surveillance System (BRFSS) for the years 2006–2010. For over two decades, the BRFSS datasets, collected via random digit telephone surveys of more than 400,000 households by the health departments of all 50 states, the District of Columbia, Guam, Puerto Rico and the US Virgin Islands have been endorsed as one of the most generalizable primary data sources for investigating numerous health conditions that contribute to morbidity and mortality in the Nation [14]. The BRFSS data sets have been widely utilized in a milieu of reports and articles. Of recent, these data have been used in studies associated with cardiovascular heart diseases [15], physical activity [16], cancer screenings [17], mental health [18] obesity [19], diabetes mellitus [20], and multiple preventive health behaviors [21, 22].

### Population sample

A population-based female sample of 1,291,803 (62%) from the BRFSS annual survey data 2006–2010, were used for these analyses (males were excluded for these analyses due to the focus on females). The sample population included 18–64 year olds across racial categories. To assess diagnoses of diabetes over the 5 year period, we concatenated datasets by variables and year of survey. All male respondents were excluded, as well as female respondents with “missing” and “refused” responses. Of the total 1,291,803 selected in this population there were 123, 385 (10%) excluded due to missing or refused responses. Though proportionally small, due to the size of the missing data there were significant differences between those with complete data and those with incomplete data. However, the proportional differences remained small. The investigators sought National University Institutional Review Board review and were determined to be exempt based on secondary data analyses on public use data [23].

### Variables

The outcome of interest was a self-reported diagnosis of diabetes. Participants in this study were indicated with diabetes with affirmation of the question, “had ever been told by a doctor that they had diabetes”. Participants who had been told that they had diabetes only during pregnancy and those reporting prediabetes or borderline diabetes were classified as not having diabetes.

The variable age was represented by the age groups in years: 18–24, 25–34, 35–44, 45–54, 55–64, and 65 and over. Exercise was measured as “physical activity or exercise in the last 30 days other than their regular job” indicated as “yes” or “no”. Body mass index was defined as weight in kilograms divided by the square of the respondent’s height in meters (kg/m^2^) and grouped into adults with body mass index at or below 25 (BMI ≤ 25) and adults with body mass index greater than 25 (BMI > 25). Race was categorized as white non-Hispanic, black non-Hispanic, Hispanic, and other non-Hispanic ethnicities. The variable year was created by indicating which year of the BRFSS the data point was associated with (2006–2010).

### Statistical analysis

Univariate analyses including chi-square tests were used to test for an association between body mass index (BMI), age, race and year of survey response. Multivariable logistic regression was used to estimate the odds of association between BMI and diagnoses of diabetes, independent of other variables. Logistic regression was also used to determine a net-effect of multiple variables on diabetes. Statistical significance was measured with 95% confidence intervals and p-values < 0.05. SAS software, version 9.2, was used for data management and statistical analyses (SAS Institute, Inc., Cary, North Carolina).

## Results

Data were complete for 1,168,418 women responders of the BRFSS 2006–2010 survey assessment (Table 1). Women responders with a BMI greater than 25 were proportionately more likely to be older, white, and less engaged in physical activity or exercise than those with a BMI at or below 25 (Table 2). Other than 2008, BMI steadily increased across age groups and across the years from 2006–2010.

**Table 1:** **Crude population characteristics**

Variable	Population
	1,168, 418 (56.0)
**BMI** ^**a**^	
BMI = 25	488,302 (41.8)
BMI > 25	680,116 (58.2)
**Age, years**	
18 to 24	36,758 (3.0)
25 to 34	113,221 (10.0)
35 to 44	169,986 (15.0)
45 to 54	232,882 (20.0)
55 to 64	246,857 (21.0)
65 or older	368,714 (32.0)
**Exercise** ^**b**^	
Yes	840, 501 (72.0)
No	327, 917 (28.0)
**Race**	
Non Hispanic White	922, 069 (79.0)
Non Hispanic Black	103, 507 (9.0)
Hispanic	81, 471 (7.0)
Other (non-Hispanic ethnicities)	61, 371 (5.0)
**Year**	
2006	197, 638 (17.0)
2007	243, 501 (20.0)
2008	233, 633 (20.0)
2009	242, 148 (21.0)
2010	251, 518 (22.0)

^a^Calculations based on self-report height and weight measures.

^b^Physical activity other than their regular job in the past 30 days.

**Table 2:** **Characteristics of survey respondents by Body Mass Index (BMI)**

Variable	Body mass index ^a^	Body mass index	***p***value*
	(BMI) ≤ 25	(BMI) > 25	
	488,302 (42.0)	680,116 (58.0)	
**Age, years**			
18 to 24	21,368 (4.0)	15,390 (2.0)	<0.0001
25 to 34	53, 752 (11.0)	59, 469 (9.0)	
35 to 44	76, 210 (16.0)	93, 776 (14.0)	
45 to 54	93, 381 (19.0)	139, 501 (21.0)	
55 to 64	88, 360 (18.0)	158, 497(23.0)	
65 or older	155, 231 (32.0)	213, 483 (31.0)	
**Exercise** ^**b**^			
Yes	382, 467 (78.0)	458, 034 (67.0)	<0.0001
No	105, 835 (22.0)	222, 082 (33.0)	
**Race**			
Non Hispanic White	53, 752 (19.0)	59, 469 (17.0)	<0.0001
Non Hispanic Black	76, 210 (28.0)	93, 776 (26.0)	
Hispanic	93, 381 (34.0)	139, 501 (40.0)	
Other (non-Hispanic ethnicities)	53, 752 (19.0)	59, 469 (17.0)	
**Year**			
2006	86,270 (18.0)	111, 368 (16.0)	
2007	102, 254 (21.0)	141, 247 (21.0)	
2008	97, 641(20.0)	135, 992 (20.0)	
2009	99, 456 (20.0)	142, 672 (21.0)	
2010	102. 681 (21.0)	148, 837 (22.0)	<0.0001

*p values based on Pearson chi-square test of association.

^a^Calculations based on self-report height and weight measures.

^b^Physical activity other than their regular job in the past 30 days.

Analyses of diabetes also showed a steady increase over the study period. Respondents without a diabetes diagnosis were proportionately more likely to be younger, report a normal weight, and report engaging in physical activity or exercise than those diagnosed with diabetes (Table 3). Significant associations between exercise and BMI (p-value = <0.0001), and for diabetes and exercise (p-value = 0.032) were noted. The results show statistically significant unadjusted associations between BMI and age, physical activity and race (*p*-values <0.0001) as well as between diabetes and age, and physical activity and race (*p*-values <0.0001).

**Table 3:** **Univariate associations of sample characteristics by diabetes**

Variable	Diagnosis of diabetes ^a^	No diagnosis of diabetes	
	130, 532 (11.2)	1, 037, 886 (88.8)	***p***value*
**BMI** ^**b**^			
BMI = 25	21, 843 (16.7)	466, 459 (44.9)	<0.0001
BMI > 25	108, 689 (83.3)	571, 427(55.1)	
**Age, years**			
18 to 24	432 (0.3)	36,326 (3.5)	<0.0001
25 to 34	2, 589 (2.0)	110,632 (10.7)	
35 to 44	7, 519 (5.8)	162,467 (15.7)	
45 to 54	19, 493 (14.9)	213,389 (20.6)	
55 to 64	35, 269 (27.0)	211,588 (20.4)	
65 or older	65, 230 (50.0)	303,484 (29.2)	
**Exercise** ^**c**^			
Yes	72, 466 (55.5)	768, 035 (74.0)	<0.0001
No	58, 066 (44.5)	269, 851 (26.0)	
**Race**			
Non Hispanic White	91,180 (69.9)	830, 889 (80.1)	<0.0001
Non Hispanic Black	19, 805 (15.2)	83, 702 (8.1)	
V Hispanic	11, 383 (8.7)	70, 088 (6.8)	
Other (non-Hispanic ethnicities)	8, 164 (6.3)	53, 207 (5.1)	
**Year**			
2006	19, 250 (14.7)	178, 388 (17.2)	
2007	26, 287 (20.1)	217, 214 (20.9)	
2008	25, 760 (19.7)	207, 873(20.0)	
2009	28, 292 (21.7)	213, 836 (20.6)	
2010	30, 943 (23.7)	220, 575 (21.3)	<0.0001

*p values are based on Pearson chi-square test of association.

^a^Self-report of diabetes mellitus diagnosed by a doctor.

^b^Calculations based on self-report height and weight measures.

^c^Physical activity other than their regular job in the past 30 days.

Table 4 represents the adjusted odds of diabetes by BMI exposure and risk factors. Minority populations were more likely to be diagnosed with diabetes than whites (n = 91,180), with blacks at a two-fold increase in odds. When compared to non-Hispanic White participants, the odds of non-Hispanic Black participants was twice as high (n = 19,805, CI = 2.15-2.23) of being told that they had diabetes, and was lower but was nearly twice as high for Hispanics (n = 11,383, CI = 1.84-1.92) and other ethnicities (n = 8,164, CI = 1.82-1.91), after adjusting for age and participants’ involvement in physical activity. Investigating by age, diabetes increased with the odds doubling after age 35. Among those diagnosed with diabetes, 108,689 (83.3%) had a BMI greater than 25.

**Table 4:** **Logistic regression analysis comparing the odds of diabetes mellitus in target population, adjusting for age, race, exercise, and year**

Variable	Diagnosis of diabetes ^a^	No diagnosis of diabetes	OR* 95% CI
	130, 532 (11.2)	1, 037, 886 (88.8)	
**BMI** ^**b**^			
BMI = 25	21, 843 (16.7)	466, 459 (44.9)	1
BMI > 25	108, 689 (83.3)	571, 427(55.1)	3.57 (3.52-3.63)
**Age, years**			
18 to 24	432 (0.3)	36,326 (3.5)	1
25 to 34	2, 589 (2.0)	110,632 (10.7)	1.82 (1.64-2.02)
35 to 44	7, 519 (5.8)	162,467 (15.7)	3.65 (3.31-4.02)
45 to 54	19, 493 (14.9)	213,389 (20.6)	7.16 (6.50-7.88)
55 to 64	35, 269 (27.0)	211,588 (20.4)	13.11 (11.91-14.43)
65 or older	65, 230 (50.0)	303,484 (29.2)	18.46 (16.77-20.31)
**Exercise** ^**c**^			
Yes	72, 466 (55.5)	768, 035 (74.0)	1
No	58, 066 (44.5)	269, 851 (26.0)	1.74 (1.72-1.76)
**Race**			
Non Hispanic White	91,180 (69.9)	830, 889 (80.1)	1
Non Hispanic Black	19, 805 (15.2)	83, 702 (8.1)	2.19 (2.15-2.23)
Hispanic	11, 383 (8.7)	70, 088 (6.8)	1.88 (1.84-1.92)
Other (non-Hispanic ethnicities)	8, 164 (6.3)	53, 207 (5.1)	1.87 (1.82-1.91)
**Year**			
2006	19, 250 (14.7)	178, 388 (17.2)	1
2007	26, 287 (20.1)	217, 214 (20.9)	1.07 (1.04-1.09)
2008	25, 760 (19.7)	207, 873(20.0)	1.05 (1.03-1.07)
2009	28, 292 (21.7)	213, 836 (20.6)	1.08 (1.06-1.10)
2010	30, 943 (23.7)	220, 575 (21.3)	1.10 (1.08-1.13)

*Adjusted odds ratios and 95% confidence intervals are from multivariable logistic regression models.

^a^Self-report of diabetes mellitus diagnosed by a doctor.

^b^Calculations based on self-report height and weight measures.

^c^Physical activity other than their regular job in the past 30 days.

Respondents with a BMI greater than 25 were almost four times more likely to have been diagnosed with diabetes than those with a BMI of 25 or less (OR = 3.57, CI = 3.51-3.63). A temporal analysis identified an approximate 2% average rise in odds of diabetes from 2006 to 2010 independent of other risk factors accounted for in this study.

## Discussion

In previous studies, upward trends in diabetes diagnoses have been mainly attributed to the rising prevalence of obesity and the lack of adequate physical activity [12, 24]. The mean proportion for BMI greater than 25 during the five year study period was 58%. This percentage is consistent with national reports of overweight and obesity. More than 60% of Americans were overweight or obese between 1990 and 2008 [25, 26] with rates leveling off to about 55% in 2009–2010 [27]. Presently, a third of the U.S population is obese [28], and it is projected that by 2030, 50% of Americans will be obese [29]. Although women (especially those of ethnic minority) are most affected by obesity and diabetes [30], there is a paucity of information over time periods exclusively among women. According to Narayan [31], individuals diagnosed with diabetes have the largest reductions in life expectancies across gender (14.3 life years in women vs. 11.6 life years for men), age, and ethnicity. Diabetes risk factors in Narayan’s study – age, ethnicity, SES, obesity and lifestyle factors, are very similar to those used in this study. While age, ethnicity, inadequate exercise and increased BMI contribute significantly to the incidence of diabetes, we ascertained the increase in odds of reported diabetes independent of these risk factors with year of survey response. This upward trend, independent of BMI and physical activity, may be attributable to advances in treatment allowing for longer survival for those diagnosed with diabetes, as well as indicate advances in diagnosis which may reduce previously misclassified disease or identify cases earlier in the disease pathway. This adjusted two percent average rise in odds of diabetes reporting from 2006–2010 allowed for a prediction of the odds of diabetes diagnoses in 2020 to be 30 percent (1.30 times higher from 2006) (OR = 1.30; CI = 1.28-1.43) if trends were persistent and modifiable and non-modifiable risk factors were held constant.

While much study has been done at the population level, diabetes has particular relevance for women, who may experience diverse societal/cultural barriers, inadequate physical activity, familial provision, and morbidity risk differently than men. These factors, both independently and in concert with each other, exert grave health effects, thus potentially contributing to health disparities at the population level. This study confirmed the association of BMI with diabetes independent of race, age, and physical activity among female participants of a large nationally representative sample. BMI is widely used as an indicative measure of being overweight or obese and is increasingly considered a proxy for chronic or delayed disease [32, 33]. Findings from this study indicate that older age, less exercise, and a BMI > 25 are associated with increased odds of clinician-diagnosed diabetes in women. For women in particular, the burden of disease is largely determined by age structure, ethnicity and SES. Higher disease rates have been observed among less affluent middle aged women and in elderly women 65 and older with a greater SES [34]. Disease rates are also impacted by urbanization and environment. There is need for ongoing surveillance of contributing risks, aimed at minimizing the toll of diabetes on those diagnosed and affected with diabetes. We observed that women with a normal BMI were at three times lower odds of being diagnosed with diabetes than women who were overweight or obese (BMI > 25). Interestingly, although Caucasian women had higher BMIs, more non-Caucasian women reported being diagnosed with diabetes. Social and ethnic-related determinants and genetics may explain the reported increase of diabetes in non-Caucasian women, compared to Caucasian women [35].

The obesity profile of the United States is changing with growing concern of becoming more racially and ethnically diverse [13] and gender focused [36, 37]. Previous reports highlight disparate effects of diabetes among minority populations [32, 33, 38] and individuals/families threatened by a lack of health insurance, and overt exposure to environmental influences. Women are simultaneously confronted with diabetes-related stressors [34], and the impact of this strain on their physical and emotional health. Our findings show that older and non-white women were more likely to report having been diagnosed with diabetes. In an attempt to see the change in odds over the years independent of other known risk factors, our analyses were adjusted for these demographics. However, interactions were not investigated for. An investigation of BMI, race, exercise, and age over the time period was conducted and did not identify a modification of effect by those variables (p-value <0.05 after Bonferonni adjustment for multiple comparisons). However, it is known that the associations of obesity with physical inactivity, age, socioeconomic status, ethnicity and body weight in populations are dynamic and complex [39]. For example, in this analysis disease-risk was higher in women with BMI over 25, and tended to be linked to ethnicity of survey respondents. This corroborates findings that we are witnessing an aging cross-cultural population that is increasing in BMI. This presents a growing challenge as we have seen from the objective of this analysis that the odds of diabetes will grow in the context of a constant risk factor set for diabetes.

Park and Kim [40], indicate that diabetes related self-efficacy in elderly minority women is influenced negatively by stressors, and positively by social support networks. In other words, increased diabetes in these women might be due to their lessened ability to make effective decisions concerning their health, and fewer people in their support networks. In addition, older women are less likely to exercise than younger women, despite known benefits of late-life exercise [41, 42].

Given the projection of diabetes in this study, health systems can assume several roles in curtailing the increased burden of diabetes, such as developing interventions through informed research, fostering collaborations through the participatory engagement of community and professional organizations, whilst being mindful of the needs of the patient and circumstances that influence health decisions.

### Limitations

Several limitations to this study should be noted. First, diabetes was determined using self-reported data and was not confirmed by physician diagnosis or medical record review. While self-report of physician diagnosed diabetes has been shown to be ideally reliable in population studies where fasting plasma glucose levels are mostly unavailable [43], there is potential for misclassification of disease which may lead to a misrepresentation of disease in this population because the diagnoses of diabetes did not distinguish between type 1 diabetes and type 2 diabetes [44]. However, type 2 diabetes accounts for more than 90% of diabetes cases in the United States [30] Also, the age group 18–24 might be more representative of a population age group with type 1 diabetes. This potential misclassification would likely be non-differential and bias any of the current findings towards the null. Second, findings may not be generalizable to certain states that did not measure diabetes in the BRFSS survey. For instance, the state of California only measured diabetes diagnoses in years 2006 & 2007. Thus, we cannot conclusively articulate actual increases of odds of diabetes in California. Third, the BRFSS excludes certain populations, including those without landline telephones and those residing in institutions and on military bases, and thus might not be representative of the entire U.S. population. Fourth, response rates were not exclusively calculated for the population subset (females) included in this analysis. The average overall response rates for the BRFSS surveys 2006 – 2010 was 33.6% (not excluding the female population) thus representing only one in three of the targeted population. This may impact generalizability despite widespread use of the BRFSS data sets by notable studies/researchers. Fifth, the survey design requires use of software capable of addressing design characteristics (unequal selection, stratification, non-response and demographic variations among populations) that may introduce bias when using statistical tools that do not take these factors into account. Although, we utilized software compatible with the BRFSS data, and were careful to exclude missing responses during the analyses, we did not weight these data to the inverse of the sampling or response design due to our focus on relevant increases in odds. Lastly, interview responses are potentially subject to recall and social desirability biases. More so, higher refusal rates found in telephone surveys may increase bias due to non-response [14]. This factor, enabled by language barriers (non-English and limited-Spanish proficiency), may account for the greater proportion of non-Hispanic whites (over 75%) compared to the low percentage rates of other ethnic minority respondents, as questionnaires were only developed using these languages. The BRFSS addresses these challenges by keeping phone (landline and cell phone) interviews to a reasonable length and increasing the number of adults interviewed in each state, so as to produce a more representative sample and higher quality data amidst changes in communications technology, societal behaviors, and population diversity [14]. More importantly, some segments of the population (e.g., the poor and uninsured) may be misrepresented in the study and there is the slight possibility that subjects may have been selected to participate in multiple years of the cross sectional assessments. Lastly, there were statistically significant differences between those with and without complete data. However, the practical differences of the missingness was minimal.

There are also notable strengths to these data. Most importantly, the BRFSS enables researchers to assess trends that provide preliminary insight into disease (e.g. diabetes) epidemic for populations. This study is unique in capturing characteristics of several influencing lifestyle factors including, physical activity in a large cohort of women residing in the United States. Having multiple years of standard questions allowed for an investigation of odds in the population over a 5-year period while accounting for important risk factors. Interactions were investigated for and not found to be significant. The use of the years was an attempt to control for surrogate variation over the time period and to give us an estimate of the projection of the increase in diabetes independent of these important factors.

## Conclusions

Overall, these findings of a large population of US women are consistent with general population findings corroborating findings of independent associations of BMI, age, physical activity, and race with diabetes. Of novel and practical importance in these analyses was the large relative increase in diabetes predicted for the forthcoming years independent of known risk factors. That is, even if we held the major modifiable and non-modifiable risk factors for diabetes constant, namely current physical activity, BMI, and population age, there would continue to be an increase in diabetes burden and thus there will be increases in the proportion of the population who have diabetes. This represents a looming major public health challenge, given the accompanying high medical expenditures and elevated risk of mortality and morbidity for this disease and has direct implications on the healthcare system as well as on long-term public health strategies. Such a trend as found in these analyses implies a near 30 percent rise in odds of diabetes diagnosis by the year 2020, independent of age, exercise and BMI. This increase is likely attributed to better diagnosis and treatment, as well as increased incidence, and highlights that reduction in incidence through education and prevention of modifiable risk factors remains imperative for the reduction in burden of this disease.

While much has been done to identify determinants of diabetes, further analysis is needed to fully elucidate the impact of the adjusted rise in odds of diabetes over the next decade. Only through efficient disease maintenance strategies integrated with strategies for diabetes prevention education at the individual level, can we hope to reduce the impact of diabetes on populations.

### Consent

A dataset containing public use data files was used for data analysis. This dataset contained aggregate responses which made seeking patient informed consent non-applicable as the data sets used does not involve access to identifiable private information about the persons from/about whom the data were collected.
